# Effects of Different Doses of Caffeine on Endurance Exercise Performance in the Heat

**DOI:** 10.3390/life15030478

**Published:** 2025-03-16

**Authors:** Weiliang Wu, Xifeng Tao, Huiyu Dong, Juan Yang, Yin Liang, Yuanyuan Lv, Laikang Yu

**Affiliations:** 1 Beijing Key Laboratory of Sports Performance and Skill Assessment, Beijing Sport University, Beijing 100084, China; wwwuweiliang@163.com; 2 Laboratory of Sports Stress and Adaptation of General Administration of Sport, Beijing Sport University, Beijing 100084, China; 3 Department of Strength and Conditioning Assessment and Monitoring, Beijing Sport University, Beijing 100084, China; txf19983480529@126.com (X.T.); 18811550083@163.com (H.D.); yj9264582022@163.com (J.Y.); 18076397318@163.com (Y.L.); 4 School of Physical Education, Xihua University, Chengdu 610000, China; 5 China Institute of Sport and Health Science, Beijing Sport University, Beijing 100084, China

**Keywords:** caffeine, endurance exercise performance, heat stress, respiratory function, aerobic capacity, fatigue perception

## Abstract

This study investigated the effects of different doses of caffeine (3 mg/kg BW and 6 mg/kg BW) on endurance exercise performance in the heat. Seventeen participants completed four randomized, double-blind trials: one in a normal environment (24.6 ± 1.2 °C) and three in a hot environment (33.2 ± 1.4 °C), with placebo, 3 mg/kg BW, and 6 mg/kg BW caffeine interventions. Endurance exercise time, cardiorespiratory function, and subjective fatigue perception were measured during incremental cycling tests. The results showed that high temperatures significantly reduced endurance exercise performance in the placebo (*p* < 0.001) and 3 mg/kg BW (*p* = 0.003) groups compared to the normal environment, but not in the 6 mg/kg BW group (*p* = 1.000). Both caffeine doses improved exercise time compared to placebo (3 mg/kg BW, *p* = 0.005; 6 mg/kg BW, *p* < 0.001). Caffeine ingestion enhanced pulmonary ventilation (VE), with significant increases in VE_peak_ (3 mg/kg BW, *p* = 0.032; 6 mg/kg BW, *p* = 0.006). Aerobic capacity improved, as evidenced by elevated VO_2_peak (3 mg/kg BW, *p* = 0.010; 6 mg/kg BW, *p* = 0.001) and PetO_2_ (3 mg/kg BW, *p* = 0.000; 6 mg/kg BW, *p* = 0.001). Subjective fatigue perception was significantly reduced only with 6 mg/kg BW caffeine (*p* = 0.020). In conclusion, caffeine ingestion at 3 mg/kg BW and 6 mg/kg BW effectively counteracts the negative effects of heat stress on endurance exercise performance by improving respiratory function, enhancing aerobic capacity, and reducing subjective fatigue. The 6 mg/kg BW dose demonstrated superior effects, making it a potential ergogenic aid for athletes training or competing in the heat.

## 1. Introduction

Endurance is a critical determinant of athletic performance, particularly in endurance-based sports, and often serves as a limiting factor in achieving world-class performance levels. Aerobic capacity, typically assessed by maximal oxygen consumption (VO_2_max) or peak oxygen uptake (VO_2_peak), plays a central role in endurance performance. Enhanced aerobic capacity delays the lactate threshold, reduces blood lactic acid (BLA) accumulation, and prolongs exercise duration, thereby improving endurance exercise performance [1]. Systematic aerobic training improves neuromuscular function, including increased capillary density, mitochondrial volume, and oxidase activity, while also supporting recovery and mitigating the impact of high-intensity training [2]. However, endurance performance is significantly influenced by environmental factors, particularly heat.

With global warming and frequent heatwaves, the need for athletes and coaches to address the challenges of exercising in hot environments has been heightened. High temperatures exacerbate physiological stress, increasing core and skin temperatures, shifting metabolism toward carbohydrate dependence, and reducing aerobic capacity [3]. These changes lead to premature fatigue and a decline in athletic performance, with endurance performance in trained athletes decreasing by 6% to 16% in hot conditions [4].

Caffeine, a widely used central nervous system stimulant, has gained popularity among athletes for its ergogenic effects, with up to 74% of elite athletes incorporating it into their routines [5]. Acute caffeine ingestion enhances sport-specific tasks [3], muscular endurance [4], and strength training performance [6,7]. It increases adrenaline and norepinephrine secretion, promotes fat mobilization, spares glycogen, and delays fatigue, thereby improving endurance exercise performance [8]. However, the optimal dosage of caffeine remains a critical consideration due to its potential for dependency and side effects. Previous studies suggested that doses between 3 and 9 mg/kg body weight (BW) are effective, with low (3 mg/kg BW) and moderate (5–6 mg/kg BW) doses improving cognitive and physical performance during intermittent and repetitive exercises [9]. High doses (9–11 mg/kg BW), however, may not enhance performance and can increase adverse effects [7].

The effect of caffeine on endurance exercise performance in hot environments remains contentious. While previous studies indicated potential negative effects on fluid balance and calorie storage during prolonged exercise in the heat [10], others demonstrated that caffeine improved performance similarly in both hot (33 °C) and cool (12 °C) environments [8]. For instance, 6 mg/kg BW caffeine ingestion prior to exercise at 30 °C and 50% humidity enhanced repetitive endurance performance in non-heat-adapted individuals [11]. Additionally, caffeine reduces muscle pain [12] and subjective fatigue [9] during high-intensity exercise in the heat, potentially increasing fatigue tolerance [10]. Despite these findings, research on caffeine’s dose-dependent effects on endurance exercise performance in hot environments remains limited, warranting further investigation to optimize its use for athletes competing under such conditions. Therefore, this study investigated the effects of different doses of caffeine (3 mg/kg BW and 6 mg/kg BW) on endurance exercise performance in the heat.

## 2. Materials and Methods

### 2.1. Participants

The required sample size was calculated using the G*power software version 3.1.9.4 (Düsseldorf, Germany), with an effect size of 0.25, α = 0.05, and power = 0.8 [13], yielding a minimum of 14 participants. To account for potential attrition, 17 strength-trained males (age: 21.6 ± 2.86 years; height: 182.34 ± 3.67 cm; BW: 75.69 ± 4.28 kg; training years: 6.38 ± 1.53 years) were recruited. The inclusion criteria included the following: (1) good health (self-reported); (2) ≥3 years of exercise experience; (3) no history of skeletal muscle or lower limb injuries or cardiovascular diseases; and (4) no caffeine dependence or adverse reaction to caffeine. All the participants provided written informed consent. The study protocol was approved by the Ethics Committee of Beijing Sport University, and all the procedures were carried out in accordance with the recommendations of the Declaration of Helsinki (protocol code 2023180H, 28 August 2023).

### 2.2. Study Design

A double-blind, randomized crossover design was employed. Each participant completed four experimental trials: one in a normal environment (24.6 ± 1.2 °C, 40–60% humidity) and three in a hot environment (33.2 ± 1.4 °C, 40–60% humidity), with each trial separated by 5 days to ensure washout and recovery [14]. The hot environment temperature (>33 °C) was selected based on evidence that it significantly reduces central activation and induces fatigue [4,15]. The temperature and humidity were measured using a thermos-hygrometer (Deli Group Co., Ltd., Ningbo, China). The participants were randomized to receive a placebo, 3 mg/kg BW caffeine, or 6 mg/kg BW caffeine in the hot environment trials. Caffeine was administered via chewing gum (Military Energy Gum^®^, Ford Gum and Machine Go, Akron, NY, USA), with placebo gum (Military Energy Gum^®^, Ford Gum and Machine Go, Akron, NY, USA) matched in appearance and flavor. The participants were instructed to avoid alcohol, caffeine, and strenuous exercise 24 h prior to testing, fast for 2 h before experiments, and maintain consistent dietary and sleep patterns.

### 2.3. Procedure

The participants underwent four experiments in a double-blind, randomized crossover design. The first experiment familiarized the participants with the exercise test. Formal tests included an incrementally loaded pedaling experiment in a normal environment. The participants measured non-fatigue data (height and weight) and performed a 5 min warm-up before cycling at 70 r/min, starting at 25 Watt and increasing by 20 Watt per minute until exhaustion [16]. A horizontal ergometer (Angio, Load, Groningen, The Netherlands) was used for this experiment. The participants lay supine on the power bike at 30-degree angle to the ground. They placed their feet into the pedals and secured them, with the seat adjusted to allow for full knee extension based on the length of the lower extremity. At the beginning of the test, the participants began pedaling in the supine position with both hands holding the handlebars on either side. For the warm-up, the participants pedaled at 25 watt for 5 min, followed by an incremental increase of 20 watt per minute until task failure. The participants were instructed to maintain a pedaling frequency between 70 and 80 revolutions per minute. If a participant’s pedaling frequency dropped below 70 revolutions per minute, they received a warning. A second occurrence of this resulted in task failure, and the ratings of perceived exertion (RPE) were immediately assessed.

Throughout the test, cardiopulmonary function parameters were analyzed in real time using a metabolic cart (MetaMax^®^ 3B, CORTEX, Isernhagen, Germany). The participants wore a heart rate (HR) monitor (Polar Electro Oy, Helsinki, Finland) throughout the test. To ensure proper fit, the patterned portion of the chest strap was moistened with water and placed snugly against the participant’s skin. The Cortex MetaSoft^®^ Studio Version 5.12.0 (CORTEX Biophysik GmbH, Leipzig, Germany) was used for the collection and analysis of cardiopulmonary function data. The participants received no feedback about exercise duration or physiological responses until all the trials had been concluded. Blinding was revealed to the participants only after all trials had been completed.

The participants were randomized into three groups: 3 mg/kg BW caffeine (CAF3), 6 mg/kg BW caffeine (CAF6), and placebo (PLA). Ventilation [pulmonary ventilation (VE), tidal volume (TV), breathing frequency (BF)], gas exchange [respiratory exchange ratio (RER), oxygen consumption (VO_2_), end-tidal partial pressure of oxygen (PetO_2_), end-tidal partial pressure of carbon dioxide (PetCO_2_)], and HR were monitored in real time. The tests were conducted in the morning to avoid circadian rhythm effects. Caffeine or placebo gum was chewed 5 min prior to warm-up, ensuring peak plasma caffeine levels during exercise [17]. Caffeine doses of 3 mg/kg BW and 6 mg/kg BW were selected based on evidence supporting their ergogenic effects and safety, with higher doses (9–12 mg/kg BW) associated with adverse effects such as sleep disturbances and anxiety [5]. All the tests were conducted at the China Institute of Sport and Health Science, Beijing Sport University.

### 2.4. Statistical Analysis

Data are presented as mean ± standard deviation (SD). Normality was checked using the Shapiro–Wilk test. Incremental load exercise performance, ventilation and metabolism parameters, and subjective fatigue were analyzed using repeated-measures ANOVA, with the Greenhouse–Geisser adjustment applied. The experimental data were analyzed using the IBM SPSS statistical software package (version 25.0, IBM, Chicago, IL, USA). The level of significance was set at *p* < 0.05.

Ventilation and metabolism data were averaged every 10 s during exercise and over the final 30 s at exhaustion. These parameters were analyzed using the individual isochronous method, where 10 time points (10% total time) were selected for each participant based on their worst trial outcome (placebo or caffeine). For example, if a participant’s worst trial lasted 400 s, the time points analyzed for both placebo and caffeine trials were 40 s, 80 s, 120 s, 160 s, 200 s, 240 s, 280 s, 320 s, 360 s, and 400 s.

## 3. Results

### 3.1. Effects of Caffeine Ingestion on Endurance Exercise Performance in the Heat

**Figure 1:**
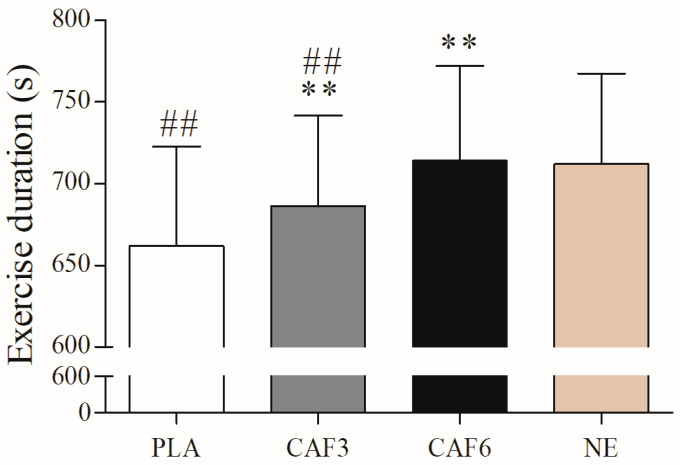
Effects of a hot environment and caffeine ingestion on endurance exercise performance. PLA, placebo group; CAF3, 3 mg/kg BW group; CAF6, 6 mg/kg BW group; NE, normal environment. Compared with NE, ## *p* < 0.01; compared with PLA, ** *p* < 0.01.

There was a significant interaction effect between group and time (F_(3,67)_ = 16.139, *p* < 0.001). As shown in Figure 1, endurance exercise time was significantly reduced in the placebo (*p* < 0.001) and 3 mg/kg BW groups (*p* = 0.003) in the hot environment compared to the normal environment, whereas no significant difference was observed in the 6 mg/kg BW group (*p* = 1.000). In the hot environment, both the 3 mg/kg BW (*p* = 0.005) and 6 mg/kg BW groups (*p* < 0.001) showed significantly longer exercise times compared to placebo. In addition, the workload at exhaustion was significantly reduced in the placebo (*p* < 0.001) and 3 mg/kg BW groups (*p* = 0.024) in the hot environment compared to the normal environment, whereas no significant difference was observed in the 6 mg/kg BW group (*p* = 1.000, Table 1).

### 3.2. Effects of Caffeine Ingestion on Respiratory Function During Endurance Exercise in the Heat

#### 3.2.1. Pulmonary Ventilation (VE)

**Figure 2:**
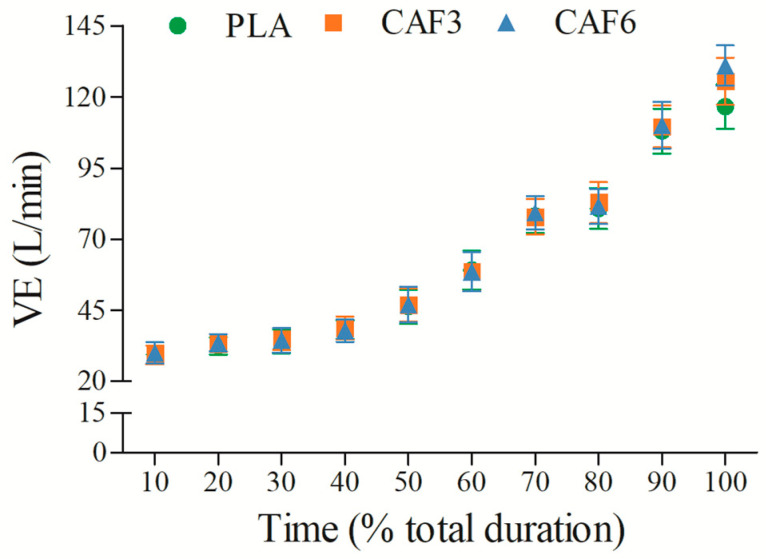
Effects of caffeine ingestion on VE during endurance exercise in the heat. PLA, placebo group; CAF3, 3 mg/kg BW group; CAF6, 6 mg/kg BW group.

VE increased progressively with exercise duration (Figure 2 and Table S1). There was a significant interaction effect between group and time (F_(2,50)_ = 10.012, *p* < 0.001). As shown in Table 2, at exhaustion, VE_peak_ was significantly higher in the 3 mg/kg BW (*p* = 0.032) and 6 mg/kg BW groups (*p* = 0.006) compared to placebo in the heat.

#### 3.2.2. Tidal Volume (TV)

**Figure 3:**
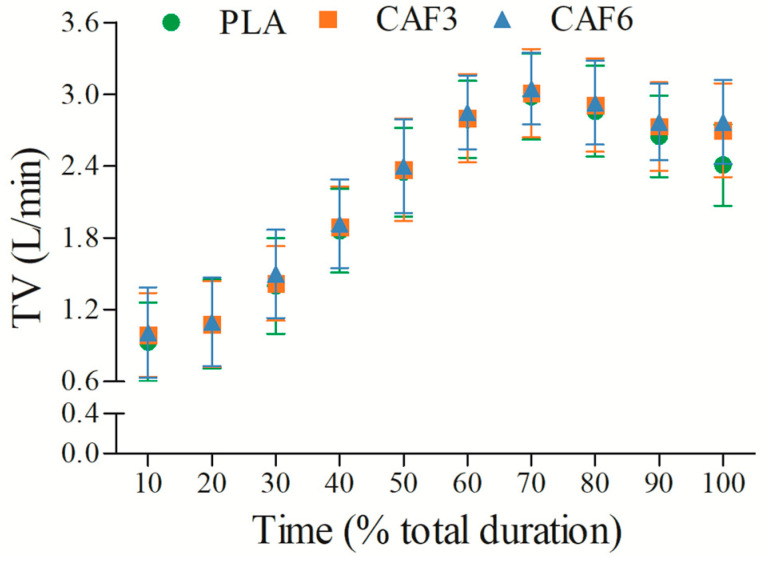
Effects of caffeine ingestion on TV during endurance exercise in the heat. PLA, placebo group; CAF3, 3 mg/kg BW group; CAF6, 6 mg/kg BW group.

TV increased gradually with exercise duration (Figure 3 and Table S1). There was a significant interaction effect between group and time (F_(2,50)_ = 12.503, *p* = 0.019). As shown in Table 2, at exhaustion, TV_peak_ was higher in the 3 mg/kg BW (*p* = 0.005) and 6 mg/kg BW groups (*p* < 0.001) compared to placebo in the heat.

#### 3.2.3. Breathing Frequency (BF)

**Figure 4:**
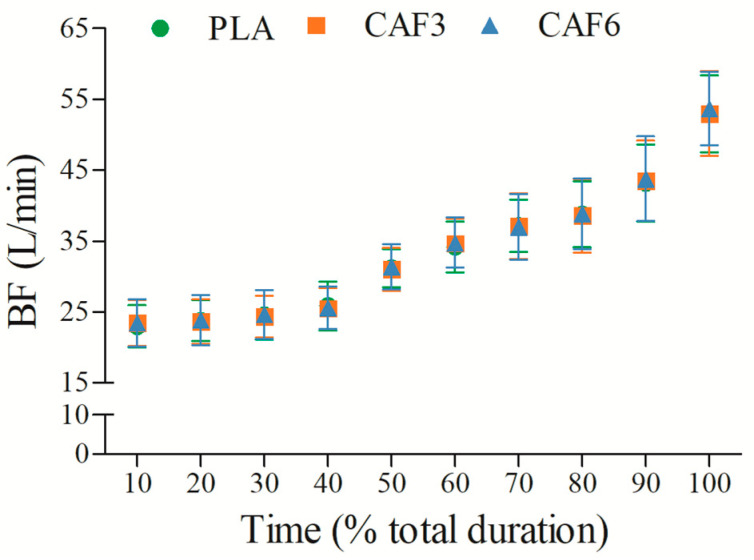
Effects of caffeine ingestion on BF during endurance exercise in the heat. PLA, placebo group; CAF3, 3 mg/kg BW group; CAF6, 6 mg/kg BW group.

BF increased progressively with exercise duration (Figure 4 and Table S1). There was a significant interaction effect between group and time (F_(2,50)_ = 3.424, *p* = 0.045). As shown in Table 2, BF_peak_ did not differ significantly between caffeine groups (3 mg/kg BW group, *p* = 1.000; 6 mg/kg BW group, *p* = 0.103) and placebo in the heat.

#### 3.2.4. Respiratory Exchange Ratio (RER)

**Figure 5:**
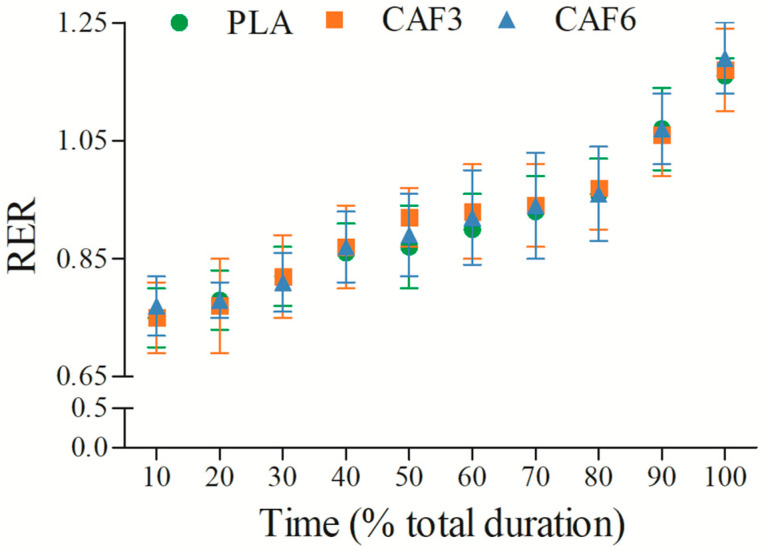
Effects of caffeine ingestion on RER during endurance exercise in the heat. PLA, placebo group; CAF3, 3 mg/kg BW group; CAF6, 6 mg/kg BW group.

RER increased gradually with exercise duration (Figure 5 and Table S1). There was a significant interaction effect between group and time (F_(2,50)_ = 12.791, *p* < 0.001). As shown in Table 2, at exhaustion, RER_peak_ was significantly higher in the 3 mg/kg BW (*p* = 0.013) and 6 mg/kg BW groups (*p* = 0.003) compared to placebo in the heat.

#### 3.2.5. Oxygen Consumption (VO_2_)

**Figure 6:**
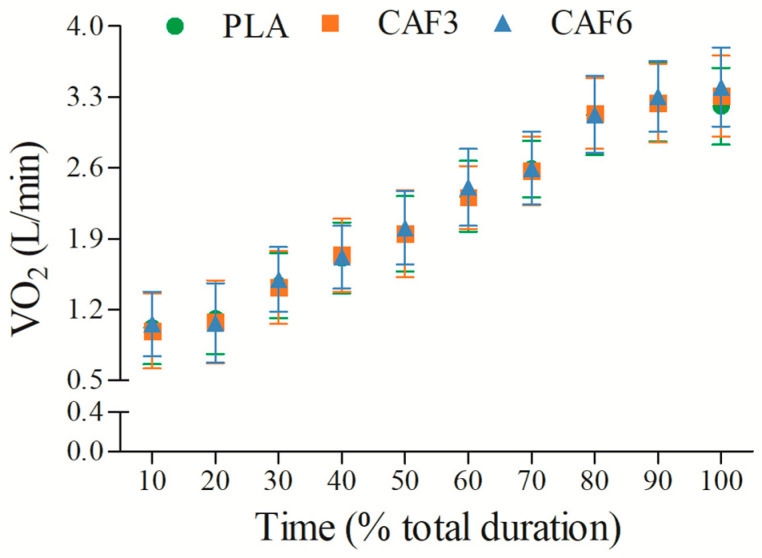
Effects of caffeine ingestion on VO_2_ during endurance exercise in the heat. PLA, placebo group; CAF3, 3 mg/kg BW group; CAF6, 6 mg/kg BW group.

VO_2_ increased progressively with exercise duration (Figure 6 and Table S1). There was a significant interaction effect between group and time (F_(2,50)_ = 14.107, *p* < 0.001). As shown in Table 2, at exhaustion, VO_2_peak was significantly higher in the 3 mg/kg BW (*p* = 0.010) and 6 mg/kg BW groups (*p* = 0.001) compared to placebo in the heat.

#### 3.2.6. End-Tidal Partial Pressure of Oxygen (PetO_2_)

**Figure 7:**
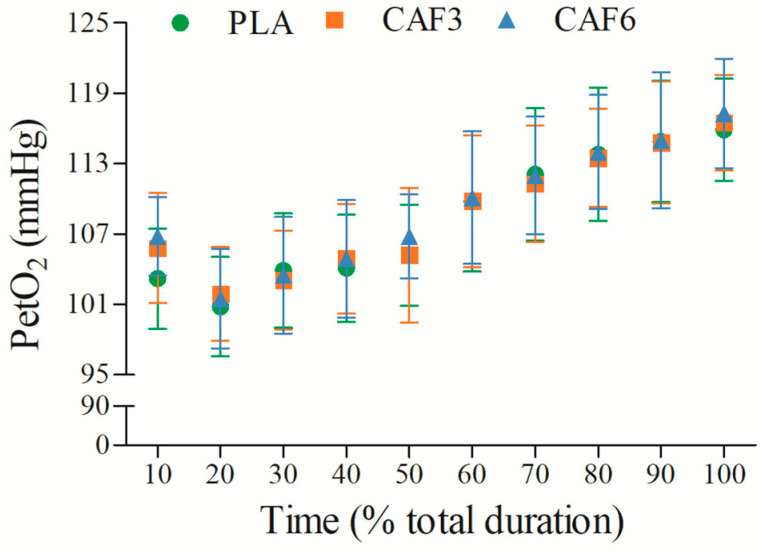
Effects of caffeine ingestion on PetO_2_ during endurance exercise in the heat. PLA, placebo group; CAF3, 3 mg/kg BW group; CAF6, 6 mg/kg BW group.

PetO_2_ initially decreased and then increased with exercise duration (Figure 7 and Table S1). There was a significant interaction effect between group and time (F_(2,50)_ = 15.631, *p* < 0.001). As shown in Table 2, at exhaustion, PetO_2_ was significantly higher in the 3 mg/kg BW (*p* < 0.001) and 6 mg/kg BW groups (*p* = 0.001) compared to placebo in the heat.

#### 3.2.7. End-Tidal Partial Pressure of Carbon Dioxide (PetCO_2_)

**Figure 8:**
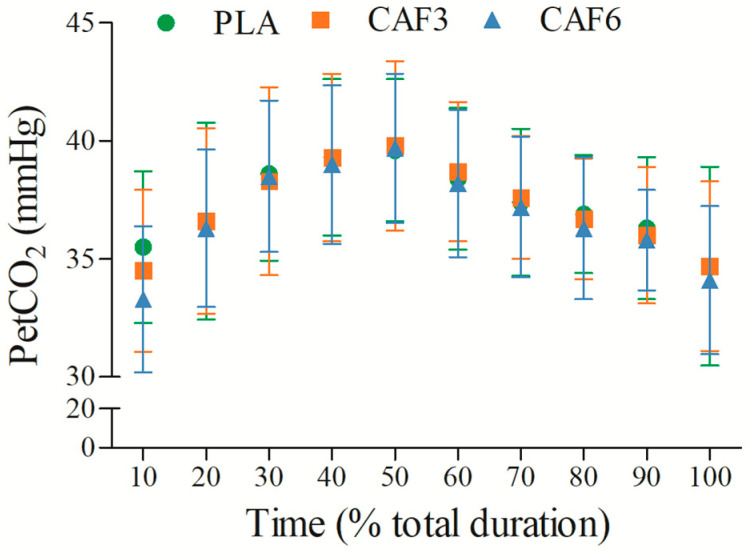
Effects of caffeine ingestion on PetCO_2_ during endurance exercise in the heat. PLA, placebo group; CAF3, 3 mg/kg BW group; CAF6, 6 mg/kg BW group.

PetCO_2_ initially increased and then decreased with exercise duration (Figure 8 and Table S1). There was no significant interaction effect between group and time (F_(2,50)_ = 1.496, *p* = 0.239). As shown in Table 2, PetCO_2_ did not differ significantly between caffeine groups (3 mg/kg BW group, *p* = 1.000; 6 mg/kg BW group, *p* = 0.233) and placebo in the heat.

### 3.3. Effects of Caffeine Ingestion on HR in the Heat

**Figure 9:**
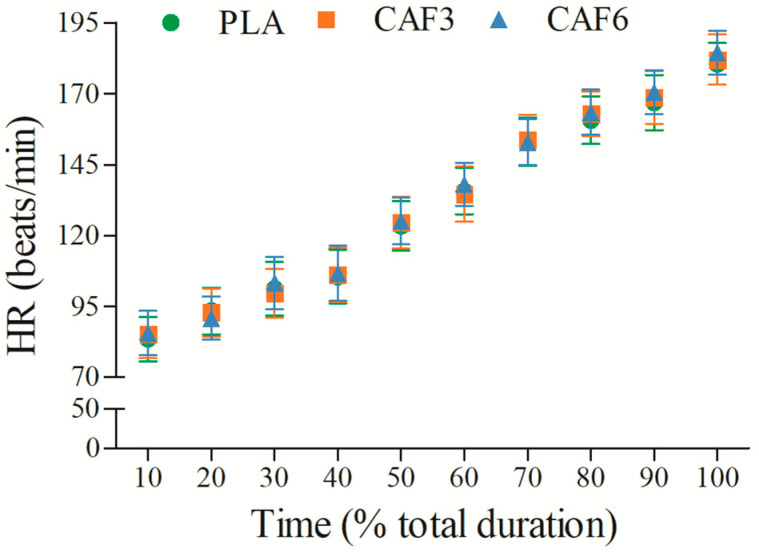
Effects of caffeine ingestion on HR during endurance exercise in the heat. PLA, placebo group; CAF3, 3 mg/kg BW group; CAF6, 6 mg/kg BW group.

HR increased gradually with exercise duration (Figure 9 and Table S1). There was a significant interaction effect between group and time (F_(2,50)_ = 4.097, *p* = 0.023). As shown in Table 2, at exhaustion, HR_peak_ was significantly higher in the 3 mg/kg BW (*p* = 0.002) and 6 mg/kg BW groups (*p* < 0.001) compared to placebo in the heat.

### 3.4. Effects of Caffeine Ingestion on RPE in the Heat

**Figure 10:**
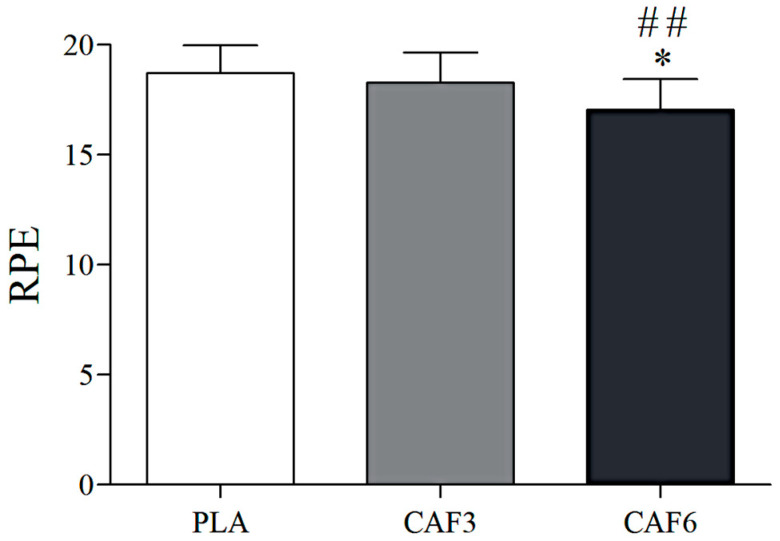
Effects of caffeine ingestion on RPE during endurance exercise in the heat. PLA, placebo group; CAF3, 3 mg/kg BW group; CAF6, 6 mg/kg BW group. Compared with PLA, * *p* < 0.05; compared with CAF3, ## *p* < 0.01.

There was a significant interaction effect between group and time (F = 7.010, *p* = 0.003). As shown in Table 2 and Figure 10, at exhaustion, RPE was significantly lower in the 6 mg/kg BW group compared to placebo in the heat (*p* = 0.020), but no significant difference was observed in the 3 mg/kg BW group (*p* = 1.000). Additionally, RPE was significantly lower in the 6 mg/kg BW group compared to the 3 mg/kg BW group (*p* = 0.001).

## 4. Discussion

This study investigated the effects of different doses of caffeine (3 mg/kg BW and 6 mg/kg BW) on endurance exercise performance in the heat. Our results demonstrated that caffeine ingestion, particularly at 6 mg/kg BW, counteracted the negative effects of heat stress on endurance exercise performance, improved respiratory function, enhanced aerobic capacity, and reduced subjective fatigue perception. These findings provide valuable insights into the ergogenic potential of caffeine under thermal stress.

### 4.1. Effects of Different Doses of Caffeine on Endurance Exercise Performance in the Heat

High temperatures significantly impaired endurance exercise performance, as evidenced by reduced exercise times in the placebo and 3 mg/kg BW groups compared to the normal environment. However, 6 mg/kg BW caffeine ingestion fully mitigated this decline, while 3 mg/kg BW partially improved performance. Previous studies have consistently shown that caffeine ingestion can enhance endurance exercise performance in the heat. For instance, one study reported significant improvements in cycling time trial performance with 3 mg/kg BW caffeine ingestion in a 35 °C heat [18], while Ganio et al. [12] observed enhanced work efficiency during 15 min cycling exercises at 33 °C with the same dose. Our findings corroborate these results, demonstrating that 3 mg/kg BW caffeine is effective in boosting endurance exercise performance in the heat. Similarly, Beaumon et al. [11] and Jones et al. [19] found that 6 mg/kg BW caffeine ingestion improved endurance exercise performance compared to placebo, further validating our observation that 6 mg/kg BW caffeine ingestion enhances endurance in hot settings.

Caffeine’s ergogenic effects are attributed to three main mechanisms: increased intracellular Ca^2+^ mobilization, accelerated free fatty acid oxidation, and antagonistic adenosine receptors in the central nervous system [20]. These mechanisms explain caffeine’s ability to improve endurance exercise performance. Most importantly, caffeine enhances endurance exercise performance through its central nervous system effects. Reduced perception of effort, a common response to caffeine ingestion, accounts for about 29% of its ergogenic effects in humans [11]. By delaying central nervous system fatigue and reducing subjective effort perception, caffeine plays a crucial role in endurance exercise performance.

Our study expanded the range of caffeine doses investigated and confirmed the consistency of its beneficial effects. Caffeine’s ergogenic properties may be attributed to its ability to cross the blood–brain barrier and bind to adenosine receptors (A1 and A2A) in the brain, reducing adenosine’s inhibitory effects. This inhibitory reduction is particularly pronounced during the initial stages of exercise, significantly potentiating endurance exercise performance that requires repetitive movements over time [21]. Moreover, caffeine ingestion significantly enhances sustained exercise performance and the mean power output of the knee extensors [22,23]. In our study, the supine pedaling exercise form relied heavily on the sustained contraction ability of the knee extensors, which correlates with endurance exercise performance and influences exercise duration. Consistent with previous research, 6 mg/kg BW caffeine ingestion improved quadriceps muscle contractility after prolonged cycling in a hot environment (36 °C) [24].

### 4.2. Effects of Different Doses of Caffeine on Respiratory Function During Endurance Exercise in the Heat

Previous studies have indicated that caffeine ingestion in hot environments alters breathing patterns by affecting respiratory rate and TV, thereby impacting pulmonary ventilation [25]. In the present study, we employed the individual isochronous method to analyze cardiorespiratory metabolism over time, providing a precise depiction of caffeine’s effects on ventilation and metabolism during incrementally loaded exercise to exhaustion. Our results showed that caffeine ingestion significantly improved pulmonary ventilation during endurance exercise in the heat. Both 3 mg/kg BW and 6 mg/kg BW doses increased TV and VE_peak_, with 6 mg/kg BW showing more pronounced effects. These findings are consistent with previous studies indicating that caffeine activates respiratory centers, elevating respiratory muscle excitatory drive [26,27], and maximal respiratory muscle strength [28].

During low-intensity exercise, TV primarily increases via inspiratory and expiratory reserves, accompanied by a moderate rise in BF. As exercise intensity rises, TV plateaus at approximately 50–60% of lung capacity, necessitating BF increases to augment VE [29]. Our study confirmed that BF significantly increased with exercise duration, with no significant difference between caffeine and placebo groups, but a tendency towards higher BF after 6 mg/kg BW caffeine ingestion, contrasting a previous study [30]. This discrepancy may stem from participant training experience. Williams et al. [30] included untrained participants, whereas our study comprised male participants > 3 years of training, exhibiting less variability in response to exercise load.

Our VE results corroborate the TV and BF findings, with significant VE elevation in the hot environment compared to placebo, regardless of caffeine dose (3 mg/kg BW or 6 mg/kg BW), more pronounced with 6 mg/kg BW. This is attributed to the following: (1) time-dependent TV and BF increases, with TV significantly higher in the caffeine group and BF tending to be higher in the 6 mg/kg BW group, leading to significant VE increases, more so with 6 mg/kg BW; (2) caffeine-induced cortical excitability and strengthened central commands during strenuous exercise, feedback-regulating peripheral stimuli, and increasing respiratory muscle work, enhancing ventilatory demand [29]. Caffeine counters respiratory muscle fatigue by blocking adenosine receptor-mediated central fatigue and exerting an antagonistic effect [31]. During moderate exercise, VE increases proportionally to metabolic rate, with caffeine-induced metabolic changes affecting VE more pronounced at 6 mg/kg BW, altering respiratory regulation and patterns [29], thereby augmenting VE and delaying exhaustion.

The results of the present study indicated that both RER and VE were significantly affected by caffeine ingestion during endurance exercise in the heat. Specifically, RER at exhaustion was notably higher in the 3 mg/kg BW and 6 mg/kg BW groups compared to placebo, with the increase more pronounced in the 6 mg/kg BW group. This finding contrasts with previous research by Marinho et al. [25] and suggests that ambient temperature and exercise duration may influence RERpeak by augmenting physiological stress and metabolic demands. The higher RER observed could reflect increased non-metabolic CO_2_ production from the blood bicarbonate buffer system, potentially exacerbated by the heat stress [3]. Furthermore, the increased metabolic stress during high-intensity exercise likely enhanced afferent signaling from respiratory and limb muscles, contributing to hyperventilation [32].

The VE results showed a significant elevation in the 6 mg/kg BW group and a trending increase in the 3 mg/kg BW group at force exhaustion compared to the placebo group. This is reinforced by the exercise time data, as both caffeine groups maintained an elevated trend without exhaustion. Similarly, TV displayed a significant trend of elevation in the caffeine groups. These findings suggest that caffeine ingestion enhances VE by improving the anti-fatigue capacity of respiratory muscle during high-intensity exercise, thereby prolonging exercise duration [33,34]. Caffeine’s mechanism of action may involve increasing central and peripheral sensitivity to CO_2_, bronchodilation, and improved respiratory muscle contractility, facilitated by its role as an adenosine receptor antagonist that stimulates sympathetic excitation [35].

### 4.3. Effects of Different Doses of Caffeine on Aerobic Capacity in the Heat

Our results showed that VO_2_ increases with exercise duration in the heat, with caffeine ingestion significantly elevating VO_2_peak at exercise exhaustion compared to placebo. This aligns with previous findings by Stadheim et al. [36], which showed caffeine enhances VO_2_peak in elite skiers, benefiting high-intensity endurance exercise performance. Although some studies reported no effect of caffeine on VO_2_peak in untrained individuals [37], our study, involving trained participants, observed a significant increase in VO_2_peak with caffeine ingestion. This effect can be attributed to improvements in maximal cardiac output (CO_peak_) and ventilatory efficiency (VE_peak_), driven by increased heart rate (HR_peak_) and stroke volume (SV) [38]. The HR_peak_ evaluation, consistent with Kovacs et al. [39], suggests caffeine enhances CO_peak_, a key mechanism for improved VO_2_peak. High temperatures may influence this effect, with prolonged exercise in the heat increasing sympathetic excitability and adrenaline secretion, further promoting energy production [40].

Our findings also support the correlation between VO_2_peak and VE_peak_ [41], with caffeine ingestion significantly increasing both VE_peak_ and PetO_2_ at exercise exhaustion, particularly with 6 mg/kg BW caffeine. This led to a more pronounced improvement in VO_2_peak in the 6 mg/kg BW group, highlighting caffeine’s role in enhancing aerobic capacity and endurance exercise performance in the heat.

Additionally, the 6 mg/kg BW group exhibited significantly higher PetO_2_ and lower PetCO_2_ at the initial time point. Caffeine ingestion is known to increase CO_2_ sensitivity at rest, thereby enhancing TV [34]. The lowering of the PetCO_2_ threshold triggers chemoreflex control of TV, leading to increased TV post-caffeine ingestion [42]. While the mechanisms controlling respiratory drive during non-fatiguing phases are not fully understood, caffeine’s ability to increase corticospinal excitability may modulate respiratory patterns, contributing to improved VE and overall endurance exercise performance [42].

### 4.4. Effects of Different Doses of Caffeine on Subjective Fatigue Perception During Endurance Exercise in the Heat

Our results indicated that caffeine ingestion at 6 mg/kg BW significantly reduced RPE at exhaustion, aligning with the results of Stadheim et al. [43]. However, the 3 mg/kg BW group showed no significant difference from the placebo; this may be attributed to the synergistic effect of a specific dose of caffeine on the human body, leading to a reduction in central nervous system fatigue, which in turn results in a more pronounced effect at higher doses. Heat stress combined with exercise intensifies muscle pain and subjective fatigue, impacting performance in hot environments [15]. Caffeine may alleviate this discomfort by boosting plasma β-endorphin levels, thereby reducing pain and fatigue during high-intensity exercise [44]. Caffeine’s lipid solubility facilitates rapid brain entry [45], binding to adenosine receptors (A1 and A2A) to diminish adenosine’s inhibitory effects, lower RPE, and enhance performance [21]. Additionally, since the workload was higher at the 6 mg/kg condition when fatigued, the RER was also higher, which may be another reason for the observed lower RPE under the 6 mg/kg condition.

Interestingly, Suvi et al. [46] showed that caffeine reduced perceived exertion and fatigue in males but not females. Similarly, Adan et al. [47] demonstrated that caffeine affects males more than females because anxiety sensitivity has been found to moderate negative psychological responses to caffeine [48]. Additionally, they suggested that females, as individuals often exhibiting high anxiety sensitivity, may influence the physical responses resulting from caffeine ingestion and affect behavioral activity in a more negative manner. Furthermore, these moderating effects of anxiety sensitivity may explain the observed effects of caffeine ingestion on the central nervous system, as well as the potentiating effects of caffeine ingestion and the negative correlation between female performance and endurance in the heat [46].

### 4.5. Strength and Limitations

Our study provides insights into the effects of caffeine on endurance exercise performance in the heat. The results show significant improvements in endurance exercise performance, respiratory function, and perceived exertion. Notably, the standard deviations associated with these metrics highlight the variability in individual responses to caffeine supplementation. This variability suggests that while caffeine generally enhances performance, individual differences in physiology, training status, and caffeine metabolism may influence the magnitude of the effect.

These findings emphasize the importance of personalized approaches in sports nutrition and performance enhancement. Future research should explore the factors that contribute to this variability, such as genetic differences in caffeine metabolism, hydration status, and individual tolerance to heat stress.

This study has some limitations. First, plasma caffeine concentrations were not measured, preventing an analysis of individual variability in caffeine metabolism. Genetic differences in caffeine metabolism (e.g., CYP1A2 and ADORA2A genotypes) may influence ergogenic responses and warrant further investigation [49]. Second, the study focused on male participants with training experience, limiting the generalizability of the findings to other populations. Future studies should include female participants and untrained individuals to explore potential sex- and training status-related differences.

## 5. Conclusions

Caffeine ingestion at 3 mg/kg BW and 6 mg/kg BW effectively counteracts the negative effects of heat stress on endurance exercise performance by improving respiratory function, enhancing aerobic capacity, and reducing subjective fatigue. The 6 mg/kg BW dose demonstrated superior effects, making it a potential ergogenic aid for athletes training or competing in the heat. However, individual variability in caffeine metabolism and response should be considered when recommending caffeine supplementation. Future research should explore the underlying mechanisms and optimal dosing strategies for caffeine use in thermal stress conditions.

## Figures and Tables

**Table 1:** The average workload at exhaustion under each condition (watt).

PLA	CAF3	CAF6	NE
245.61 ± 20.27 ##	253.71 ± 18.49 #**	263.00 ± 19.34 **	262.31 ± 18.48

Note: PLA, placebo group; CAF3, 3 mg/kg BW group; CAF6, 6 mg/kg BW group; NE, normal environment. Compared with NE, # *p* < 0.05, ## *p* < 0.01; compared with PLA, ** *p* < 0.01.

**Table 2:** Effects of caffeine ingestion on cardiopulmonary function during endurance exercise exhaustion in the heat.

Groups	PLA	CAF3	CAF6
VE	116.6 ± 11.9	129.1 ± 11.2 *	133.5 ± 11.5 **
TV	2.41 ± 0.34	2.66 ± 0.37 **	2.75 ± 0.35 **
BF	53.0 ± 5.44	53.2 ± 5.77	54.0 ± 6.69
RER	1.16 ± 0.04	1.20 ± 0.05 *	1.24 ± 0.06 **
VO_2_	3.21 ± 0.38	3.45 ± 0.30 *	3.53 ± 0.29 **
PetO_2_	115.9 ± 4.34	118.8 ± 4.17 **	120.1 ± 4.52 **
PetCO_2_	34.7 ± 3.21	34.4 ± 3.57	33.3 ± 3.68
HR	180.5 ± 7.4	186.4 ± 7.5 **	187.3 ± 7.6 **
RPE	18.71 ± 1.27	18.30 ± 1.36	17.06 ± 1.40 *##

Note: PLA, placebo group; CAF3, 3 mg/kg BW group; CAF6, 6 mg/kg BW group; VE, pulmonary ventilation; TV, tidal volume; BF, breathing frequency; RER, respiratory exchange ratio; VO_2_, oxygen consumption; PetO_2_, end-tidal partial pressure of oxygen; PetCO_2_, end-tidal partial pressure of carbon dioxide; HR, heart rate; RPE, ratings of perceived exertion. Compared with PLA, * *p* < 0.05 and ** *p* < 0.01; compared with CAF, ## *p* < 0.01. The data were generated over the final 30 s at exhaustion.

## Data Availability

All the data generated or analyzed during this study are included in the article/Supplementary Materials.

## Supplementary Materials

The following supporting information can be downloaded at https://www.mdpi.com/article/10.3390/life15030478/s1, Table S1: Effects of caffeine on respiratory function and heart rate during endurance exercise in the heat.
