# Supplementary material for: Effects of Different Doses of Caffeine on Endurance Exercise Performance in the Heat
Source: Life (Basel). 2025 Mar 16;15(3):478. doi: 10.3390/life15030478 (PMC11943937; doi:10.3390/life15030478)
Supplement: Supplementary file 1 [file life-15-00478-s001.zip › life-3514931-supplementary.pdf]

Table S1. Effects of caffeine on respiratory function and heart rate during endurance exercise in the heat.

	Groups	10% ET	20% ET	30% ET	40% ET	50% ET	60% ET	70% ET	80% ET	90% ET	100% ET
	PLA	29.5 ± 2.67	32.5 ± 3.05	34.2 ± 4.22	38.2 ± 3.31	46.4 ± 6.02	59.2 ± 6.78	78.2 ± 5.92	80.8 ± 7.15	108.5 ± 7.88	116.6 ± 7.74
VE	CAF3	29.3 ± 3.25	33.4 ± 2.89	35.0 ± 3.89	38.8 ± 4.01	46.9 ± 5.90	58.7 ± 6.77	77.9 ± 6.22	82.9 ± 7.23	109.6 ± 7.35	125.0 ± 8.32
	CAF6	30.1 ± 3.73	33.6 ± 3.01	34.6 ± 4.33	37.9 ± 3.99	47.0 ± 6.29	58.6 ± 6.89	79.3 ± 5.85	81.6 ± 6.21	110.1 ± 8.23	131.0 ± 7.09
	PLA	0.93 ± 0.33	1.08 ± 0.37	1.40 ± 0.40	1.86 ± 0.35	2.35 ± 0.37	2.79 ± 0.32	2.98 ± 0.36	2.86 ± 0.38	2.65 ± 0.34	2.41 ± 0.34
TV	CAF3	0.99 ± 0.35	1.08 ± 0.36	1.42 ± 0.31	1.89 ± 0.34	2.37 ± 0.43	2.80 ± 0.37	3.01 ± 0.37	2.91 ± 0.39	2.73 ± 0.37	2.70 ± 0.39
	CAF6	1.01 ± 0.38	1.10 ± 0.37	1.50 ± 0.37	1.92 ± 0.37	2.40 ± 0.39	2.85 ± 0.31	3.05 ± 0.30	2.93 ± 0.35	2.74 ± 0.32	2.77 ± 0.35
	PLA	23.0 ± 2.98	23.8 ± 2.89	24.6 ± 3.49	25.4 ± 3.44	31.2 ± 2.68	34.2 ± 3.61	37.2 ± 3.69	38.8 ± 4.69	43.2 ± 5.41	53.0 ± 5.44
BF	CAF3	23.5 ± 3.25	23.7 ± 3.15	24.4 ± 2.94	25.5 ± 2.89	31.2 ± 3.01	34.7 ± 3.46	37.1 ± 4.65	38.6 ± 5.21	43.5 ± 5.67	53.0 ± 6.01
	CAF6	23.5 ± 3.32	23.9 ± 3.56	24.7 ± 3.46	25.6 ± 2.98	31.4 ± 3.21	34.9 ± 3.56	37.4 ± 4.64	38.9 ± 5.01	43.8 ± 5.97	53.7 ± 5.21
	PLA	0.75 ± 0.05	0.78 ± 0.05	0.82 ± 0.05	0.86 ± 0.05	0.87 ± 0.07	0.90 ± 0.06	0.93 ± 0.06	0.96 ± 0.06	1.07 ± 0.07	1.16 ± 0.04
RER	CAF3	0.75 ± 0.06	0.77 ± 0.08	0.82 ± 0.07	0.87 ± 0.07	0.92 ± 0.05	0.93 ± 0.08	0.94 ± 0.07	0.97 ± 0.07	1.06 ± 0.07	1.17 ± 0.07
	CAF6	0.77 ± 0.05	0.78 ± 0.03	0.81 ± 0.05	0.87 ± 0.06	0.89 ± 0.07	0.92 ± 0.08	0.94 ± 0.09	0.96 ± 0.08	1.07 ± 0.06	1.19 ± 0.06
	PLA	1.02 ± 0.36	1.11 ± 0.35	1.44 ± 0.32	1.71 ± 0.35	1.95 ± 0.37	2.32 ± 0.35	2.59 ± 0.28	3.12 ± 0.39	3.25 ± 0.39	3.21 ± 0.38
VO_2	CAF3	0.99 ± 0.37	1.08 ± 0.41	1.42 ± 0.36	1.74 ± 0.36	1.95 ± 0.43	2.31 ± 0.31	2.57 ± 0.34	3.14 ± 0.35	3.24 ± 0.39	3.31 ± 0.40
	CAF6	1.06 ± 0.32	1.07 ± 0.39	1.50 ± 0.32	1.72 ± 0.31	2.01 ± 0.36	2.41 ± 0.38	2.60 ± 0.36	3.13 ± 0.38	3.31 ± 0.35	3.40 ± 0.39
	PLA	103.2 ± 4.26	100.8 ± 4.25	103.9 ± 4.87	104.1 ± 4.56	105.2 ± 4.31	109.8 ± 5.98	112.1 ± 5.64	113.8 ± 5.69	114.6 ± 5.19	115.9 ± 4.34
$PetO_2$	CAF3	105.8 ± 4.69	101.9 ± 3.98	103.1 ± 4.22	104.9 ± 4.68	105.8 ± 5.73	109.8 ± 5.62	111.3 ± 4.96	113.6 ± 4.21	114.8 ± 5.21	116.5 ± 4.08
	CAF6	106.8 ± 4.23	101.5 ± 3.36	103.5 ± 4.98	104.9 ± 5.01	106.8 ± 3.56	110.1 ± 5.64	112.0 ± 5.01	114.0 ± 4.88	115.0 ± 5.79	117.3 ± 4.66
	PLA	35.5 ± 3.21	36.6 ± 4.17	38.6 ± 3.68	39.3 ± 3.31	39.6 ± 3.01	38.4 ± 3.00	37.4 ± 3.12	36.9 ± 2.49	36.3 ± 3.01	34.7 ± 4.21
$PetCO_2$	CAF3	34.5 ± 3.43	36.6 ± 3.94	38.3 ± 3.98	39.3 ± 3.56	39.8 ± 3.59	38.7 ± 2.95	37.6 ± 2.59	36.7 ± 2.56	36.0 ± 2.89	34.7 ± 3.59
	CAF6	33.3 ± 3.10	36.3 ± 3.33	38.5 ± 3.21	39.0 ± 3.36	39.7 ± 3.16	38.2 ± 3.13	37.2 ± 2.98	36.3 ± 3.01	35.8 ± 2.13	34.1 ± 3.14
	PLA	83.5 ± 7.9	93.4 ± 8.2	101.2 ± 9.4	105.5 ± 9.5	123.4 ± 8.8	135.7 ± 8.2	153.1 ± 8.5	160.7 ± 8.4	166.9 ± 9.7	180.5 ± 7.4
HR	CAF3	85.1 ± 8.4	92.9 ± 8.5	99.7 ± 8.6	106.1 ± 9.5	124.5 ± 9.2	134.6 ± 9.7	153.7 ± 8.9	162.9 ± 7.8	168.7 ± 9.3	182.0 ± 8.9
	CAF6	85.6 ± 7.8	90.9 ± 7.6	103.3 ± 9.0	106.7 ± 9.8	125.1 ± 8.2	138.1 ± 7.6	152.9 ± 8.1	163.4 ± 8.0	170.5 ± 7.7	184.5 ± 7.7

Note: PLA, placebo group; CAF3, 3 mg/kg BW group; CAF6, 6 mg/kg BW group; ET, exhaustion time; VE, pulmonary ventilation; TV, tidal volume; BF, breathing frequency; RER, respiratory exchange ratio; VO_2, oxygen consumption; $PetO_2$, end-tidal partial pressure of oxygen; $PetCO_2$, end-tidal partial pressure of carbon dioxide; HR, heart rate.
